# A case report: anaphylaxis to cefazolin during renal transplant surgery

**DOI:** 10.1186/s13223-021-00559-w

**Published:** 2021-05-29

**Authors:** Kaveh Hemati, Shelley Gierat, Garrett R. Roll, Odmara L. Barreto Chang

**Affiliations:** 1 Department of Anesthesia and Perioperative Care, University of California San Francisco , 521 Parnassus Ave, 4th Floor, San Francisco, California 94143 USA; 2 Department of Surgery, Division of Transplant, University of California San Francisco, San Francisco, California 94143 USA

**Keywords:** Anaphylaxis, Renal transplant, Transplant surgery, Cefazolin, Intraoperative, Anesthesia, Tryptase

## Abstract

**Background:**

While there exist case reports of anaphylaxis occurring during renal transplant surgery, descriptions of continuing transplant surgery post-anaphylaxis have been scarce. Anaphylactic reactions that present solely with hypotension without pulmonary or mucocutaneous signs have yet to be described during renal transplant surgery.

**Case presentation:**

Here we report a case of a 33-year-old female with end-stage renal disease who underwent cadaveric renal transplant. She developed anaphylaxis following the administration of cefazolin. Despite this reaction, the surgery was ultimately completed after patient stabilization, and the patient had excellent graft function postoperatively. The patient had an elevated tryptase at the time of the reaction and postoperative allergy testing revealed a positive intradermal test to cefazolin. Written informed consent was obtained from the patient for all procedures, studies, and publication of this case report.

**Conclusions:**

This is the first case of a successful zero-mismatch cadaveric renal transplant following an anaphylactic reaction to cefazolin. Although anaphylaxis during transplant surgery typically warrants cancellation due to the hemodynamic effects that may lead to graft dysfunction, here we describe a case where surgery was continued following patient stabilization. The decision to proceed with surgery despite an intraoperative emergency along with the management and workup of intraoperative anaphylaxis are described, which can be beneficial for others who are presented with similar scenarios in the future.

## Introduction

Intraoperative anaphylaxis is a life-threatening condition with a mortality rate of 3–10% and presents a unique challenge for the anesthesiologist in patients undergoing transplant surgery. It has been shown that the median time interval between the onset of signs and symptoms and cardiac arrest is 5 min in iatrogenic anaphylaxis [1]. Of all anaphylactic reactions, skin symptoms and signs are present in up to 90 percent of episodes while respiratory compromise is present in up to 85% [2, 3]. Although these common signs and symptoms can be helpful in the diagnosis of anaphylaxis and subsequent management, they are not always present. Patients with anaphylaxis who present with only hypotension typically have been exposed to substances to which they are known to be allergic. Here we describe a presentation of a successful renal transplant following anaphylaxis to cefazolin in a patient who had two prior exposures but no known allergy to the offending agent. Written informed consent was obtained from the patient for publication of this case report.

## Case description

A 33-year-old, 50-kg female with end-stage renal disease (ESRD) secondary to reflux nephropathy was scheduled for a zero-mismatch cadaveric renal transplant. She was dependent on peritoneal dialysis, and her past medical history was also notable for anemia and secondary hyperparathyroidism. She was on multiple antihypertensive medications, and she reported no drug allergies, which was also confirmed with a review of the medical record. Notably, however, she did verbalize a reaction to an unknown sedative that she received for a procedure in 2019 that resulted in the sole symptom of pruritus in her lower extremities. Aside from this reaction, she had several previous general anesthetics that had been uncomplicated and had received cefazolin twice in the past without adverse reactions.

The patient was hypertensive before induction of anesthesia with systolic blood pressure (SBP) range from 170 s to 200 mmHg and a diastolic blood pressure (DBP) range from 95 to 110 mmHg. Her heart rate was normal in the 70 s and oxygen saturation was greater than 95%. Induction of anesthesia was achieved with propofol and cisatracurium, and anesthesia was maintained with sevoflurane. The lowest blood pressure reading during and after induction was 137/89 mmHg, and the patient state index (PSI) on SedLine was between 10 and 30. The routine peri-transplant immunosuppressive dose of methylprednisolone (total 500 mg) was started 12 min after induction and given over 25 min. A routine bolus of cefazolin (2 g) was given intravenously (IV) over 3 min prior to incision.

The surgical team made the incision, and 4 min following the administration of cefazolin, the blood pressure decreased from 140/80 to 75/44 mmHg. No bronchospasm or mucocutaneous signs were appreciated. However, tachycardia, hypotension, and cardiovascular collapse were observed. In addition, the pleth variability index (PVI) increased significantly from 4 to 14, and PSI remained between 20 and 30. She was initially treated with phenylephrine. She was not responsive to the phenylephrine and received epinephrine (total 100 mcg). The hypotension worsened (55/45 mmHg), she had bradycardia in the 50 s, oxygen saturation in the 70 s, and the femoral pulse was not palpable. Sevoflurane and methylprednisolone were both discontinued. The SedLine raw electroencephalogram did not display burst suppression, and PSI was between 20 and 30. The five-lead electrocardiogram was assessed, and no changes were noted. The ventilator showed normal peak pressures, and the capnograph waveform shape was normal. The end-tidal carbon dioxide (ETCO2) had decreased from the high 30 s to the low 20 s. The patient’s skin and oral mucosa appeared normal and the lungs were clear bilaterally. The patient was given 1.5 mg of epinephrine followed by 18 units of vasopressin to achieve hemodynamic stability. Within 7 min, hemodynamics improved to baseline. The delivery of sevoflurane was resumed, with the highest PSI noted to be 43. A tryptase level was sent. A discussion was held about whether or not to proceed with the planned surgical procedure.

The patient was observed for approximately 20 min during which she remained stable. The decision was made to proceed with the kidney transplant due to the excellent immunologic matching between the donor and recipient (zero antigen mismatch). The surgical procedure was resumed, and the patient remained stable for the rest of the case without the need for additional vasopressors or inotropes. She was admitted to the intensive care unit (ICU) for observation secondary to presumed intraoperative anaphylaxis and a near cardiopulmonary arrest. Her ICU admission was uncomplicated except for the need for oral midodrine on postoperative day 1, which she received to achieve a mean arterial pressure (MAP) goal of > 70 to provide adequate perfusion to the renal allograft.

**Fig. 1:**
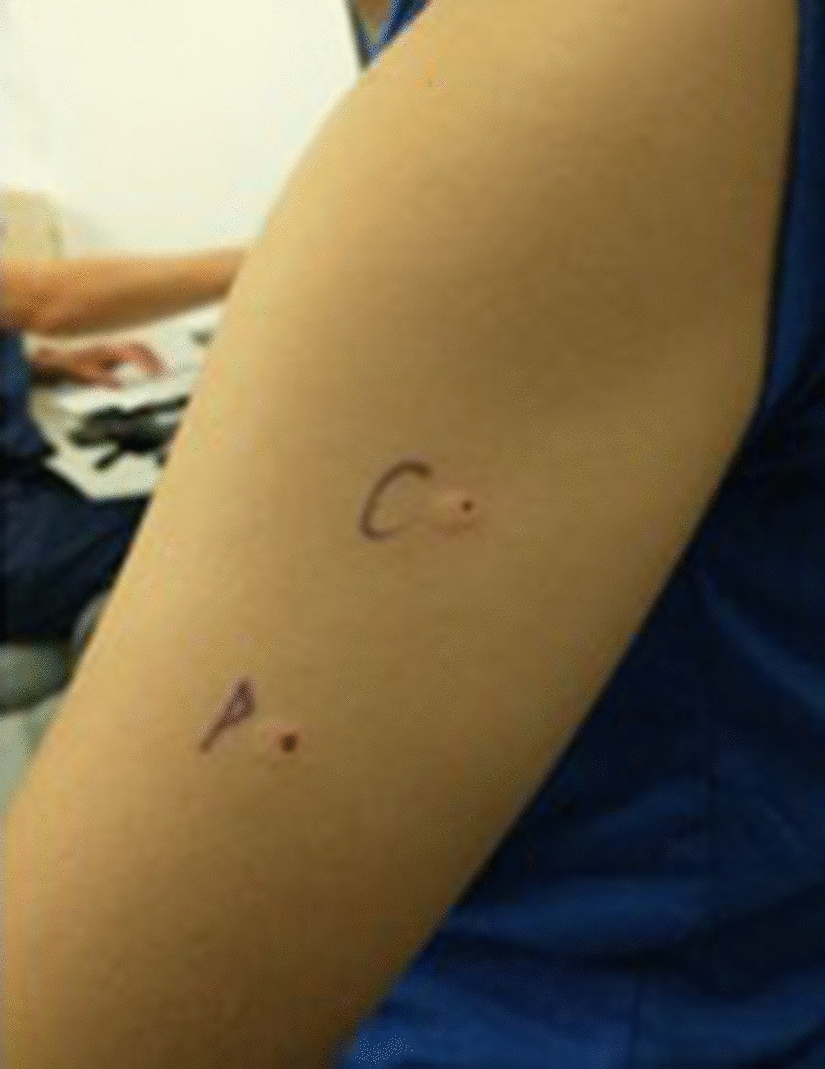
Skin testing. Cefazolin was positive on intradermal testing at 3.3 mg/ml, producing a wheal > 5 mm (C) when compared to positive control (P). Intradermal tests were conducted on other medications including penicillin, propofol, and cisatracurium. The results of these tests were negative (not shown)

On postoperative day 2, the MAPs ranged in the 80–100 s and midodrine was discontinued. The patient was transferred out of the ICU. She had excellent early graft function and her laboratory results were as expected until discharge on post-operative day 4. Her intraoperative tryptase level was elevated at 51.8 mcg/L (normal < 11 mcg/L), and 1 month following surgery returned to normal (5.5 mcg/L). As part of her intraoperative anaphylaxis work-up, the patient was seen in the Allergy and Immunology Clinic for skin prick testing of multiple agents she had been exposed to intraoperatively. Propofol, penicillin, and amoxicillin skin testing were negative. Methylprednisolone was initially considered as a potential etiologic agent, but since she received large doses subsequently on postoperative days 1 and 2 without any reaction it was excluded. Latex and chlorhexidine were not considered likely culprits given the lack of their temporal relationship between exposure and reaction. The cefazolin skin testing was positive (Fig. 1), suggesting that cefazolin was the likely cause of her intraoperative anaphylactic episode.

## Discussion and conclusions

Intraoperative anaphylaxis is a medical emergency that poses significant challenges to the anesthesiologist, especially in transplant surgery [4–6]. During general anesthesia, 60 percent of all anaphylactic reactions are triggered by neuromuscular blocking agents, followed by latex (20 percent), and antibiotics (15 percent) [7]. Penicillins and cephalosporins are responsible for approximately 70 percent of all cases of antibiotic-induced anaphylaxis [7].

Anaphylactic and anaphylactoid reactions may have indistinguishable presenting symptoms; however, they are caused by different pathophysiology. An anaphylactic reaction is an IgE-mediated allergic reaction following the massive release of mediators from mast cells as a response to an allergen [5, 8]. Anaphylactoid reactions are non-IgE mediated and occur through a direct nonimmune-mediated release of mediators from mast cells and basophils [5, 8]. The mainstay of treatment of both acute anaphylactic and anaphylactoid reactions is epinephrine [2]. Although other modalities can be used, including antihistamines, corticosteroids, and β-2 agonists, they should only be used as adjuncts to epinephrine as it has been shown that lack of or delayed epinephrine administration has led to worse outcomes and mortality [2].

This patient’s initial presentation of severe hypotension without obvious pulmonary or mucocutaneous signs raised the possibility of an anaphylactic reaction after alternative differential diagnoses were ruled out. Fluids, epinephrine, and additional vasopressor support were given to achieve hemodynamic stability. Given that the patient didn’t present with the classic features of anaphylaxis, a confirmatory tryptase level was sent and she was followed by the Allergist for skin testing.

There are several testable indicators of anaphylactic reactions, including histamine and tryptase. Tryptase is the most specific product of mast cells and basophils, and thus has become one of the more reliant tests to assess for anaphylactic reactions [9]. When anaphylaxis is considered intraoperatively, it is the anesthesiologist’s responsibility to manage the clinical situation with supportive therapies and help facilitate the diagnostic workup. If the diagnosis of anaphylaxis is not clear due to a non-traditional presentation, it is recommended to send a tryptase level to confirm or rule out the diagnosis [2, 4, 6]. Tryptase levels peak one and half hours after the onset of anaphylaxis, but levels drawn up to 3 h after symptom onset can support the diagnosis [4, 10]. Additionally, the anesthesiologist should ensure that the patient is counseled regarding the event and a referral placed to see an allergist for skin testing 1 month following the event [6].

Anaphylaxis after induction certainly warrants cancellation of elective surgery because it can be challenging to predict the hemodynamics that will occur over the subsequent few hours, and frequently the patient has vasopressor requirements lasting long after the event. To avoid exposing a renal allograft to hypotension and vasopressors post-operatively, kidney transplants are generally cancelled when the recipient has anaphylaxis. The unique aspect of this case was the immune matching between the deceased donor and recipient, making it extremely unlikely this patient would ever get another deceased donor kidney of equal quality. The surgical and anesthesia teams agreed to move forward with the transplant, to admit the patient to the ICU to watch for a possible biphasic reaction, and midodrine was started preemptively upon admission to the ICU.

Biphasic reactions, which are defined as recurrence of anaphylaxis within 72 h of the initial reaction without re-exposure to the offending agent, occur in less than 5% of patients diagnosed with anaphylaxis [11]. It may be prudent to observe patients for an adequate time-period following an acute anaphylaxis episode to monitor for a biphasic reaction, and it is recommended to monitor patients for at least 6–12 h following the event [2, 11].

The patient’s skin testing and tryptase levels supported the diagnosis of cefazolin-induced anaphylaxis. Despite an intraoperative emergency presenting in a non-traditional fashion, the patient had a successful kidney transplant and will now avoid cefazolin in the future.

## Data Availability

Data sharing is not applicable to this article as no datasets were generated or analyzed for this case report.

## Abbreviations

DBP: Diastolic blood pressure
ESRD: End-stage renal disease
ETCO2: End-tidal carbon dioxide
ICU: Intensive care unit
MAP: Mean arterial pressure
PSI: Patient state index
PVI: Pleth variability index
SBP: Systolic blood pressure
